# If You Build It, Will They Come? An Analysis of Candidate Attitudes Toward A New Residency Program

**DOI:** 10.15694/mep.2018.0000245.1

**Published:** 2018-11-05

**Authors:** Michelle Barajaz, Sarah Denniston, Shelley Kumar, Adam Wolfe

**Affiliations:** 1 Baylor College of Medicine; The Children's Hospital of San Antonio; 2 The Center for Research Innovation

**Keywords:** Recruitment, New Program, Ranking, Graduate Medical Education, Workforce

## Abstract

This article was migrated. The article was marked as recommended.

**Background:** To address looming healthcare workforce shortages, the Association of American Medical Colleges (AAMC) has recommended a substantial increase in residency positions. However, new residency programs face unique recruitment challenges.

**Objective:** To identify new program characteristics and recruitment practices that most influence candidate-ranking decisions.

**Methods:** In a post-match survey, applicants who interviewed during the first two recruitment seasons of a new program were asked to rate program characteristics and recruitment techniques regarding the effects they had on ranking decisions, and to describe the most attractive and concerning features. Somers’ *D* directional coefficients were calculated to determine the correlation between applicants’ ranking of the program and ranking of survey items. Qualitative responses were analyzed as word clouds.

**Results:** 163/349 surveys were returned (47%). The strongest correlating items included the opportunities to work closely with faculty, to help shape a new program, and to be one of the first graduates. Influential recruitment practices included program communications and website vividness. Concerns included lack of senior residents, fear of unforeseen difficulties, and no evidence of outcomes.

**Conclusion:** New programs have unique features that influence candidates’ ranking decisions. Recruitment practices should be designed to identify candidates who embrace the unique opportunities of a new program.

## Introduction

The international physician workforce is already strained to meet the needs of patients, described recently as a “global crisis” ( Sirili et al., 2017), and there have been increasing calls for expanded medical training opportunities globally ( Ibrahim et al., 2016, Sirili et al., 2017, Domagala and Klich, 2018). In the United States, the Association of American Medical Colleges (AAMC) estimates a shortage of 61,700 - 94,700 physicians by 2025 ( Dall et al., 2016). The AAMC reported in 2015 that first-year medical student enrollment is projected to grow 30% between 2002 and 2018 ( AAMC Center for Workforce Studies, 2016); however, the subsequent need for more graduate medical training positions has not been addressed. One solution is to develop 3,000 Medicare-funded resident training positions per year between 2017 and 2021 ( AAMC Center for Workforce Studies, 2016). Since most existing programs are limited in the growth they can support, new residency programs are needed and have specific logistics for being created ( Barajaz and Turner, 2016).

It can be challenging to attract applicants to a program with no proven track record. An 8-year study of applicants to programs at Massachusetts General Hospital revealed that two key attractive features were resident morale and preparation for future training/career ( Phitayakorn et al., 2015). In the 2015 National Residency Matching Program (NRMP) applicant survey, seven of the top 16 factors important to ranking decisions (reputation, resident quality, work/life balance, house staff morale, future fellowship opportunities, career paths of graduates, and preparation for fellowship training) cannot be assessed in new training programs (NRMP, 2015), and outcomes data such as board passage rates are likewise unavailable. Candidates for a new program might be apprehensive of the untried nature of the program, lack of upper-level residents, and level of institutional support. Conversely, a new program offers unique opportunities that can prove attractive (e.g., close working relationships with faculty, the ability to shape and influence a new program). Identifying these factors and determining their roles in ranking decisions can inform both the recruitment and selection processes.

Business literature offers cognitive frameworks surrounding acceptance decisions with regard to the features that attract or concern applicants. The Cornell Center for Advanced Human Resources Studies ( Rynes, 1989) offers two theories related to job selection: 1) The *Objective Factor Theory* ( Maclean and Pato, 2011) which states that applicants are influenced by the attributes of the job, how well that job is “sold” to them, and the organization’s reputation and stature, and 2) the *Critical Contact Theory,* which states that applicants are influenced by comments and behaviors of the interviewer as an organizational representative. Both frameworks demonstrate that the organization itself greatly influences the recruitment process. Hence, identifying the qualities that are most important to applicants in the unique situation of interviewing at a new program is critical to the quality of recruitment outcomes ( Schreurs and Syed, 2011). For instance, in one psychiatry program, a change in recruitment process yielded a sustained increase in fill rates and median test scores ( Maclean and Pato, 2011).

The factors that are important to applicants at a new residency program have not been reported. We surveyed interviewees at the new Baylor College of Medicine (BCM) Pediatrics Residency Program at The Children’s Hospital of San Antonio (CHofSA) during the first two recruitment seasons. This is an ACGME accredited program that accepted 10 residents per year in the first two recruitment cycles (2015 and 2016). Our primary training site is a new free-standing children’s hospital with 190 inpatient beds. It includes a 60 bed level 4 Neonatal Intensive Care Unit, a 40 bed combined Pediatric Intensive Care/Intermediate Care Unit, and a 48 bed emergency room. It is staffed with approximately 200 faculty across general pediatrics and the medical and surgical subspecialties. Applicants rated the impact of specific program characteristics and recruitment practices on their decisions to rank the program. The results revealed distinct features of a new program that strongly correlated with applicants’ ranking decisions. This study should assist leaders of new residency programs in designing materials and practices to optimize their recruitment.

## Methods

### Study participants and eligibility

Institutional Review Board approval was obtained from both BCM and Christus Santa Rosa Health System, the parent entity of CHofSA. Eligible participants interviewed at CHofSA for the 2015 and 2016 categorical pediatrics residency match. An anonymous survey was sent electronically ( https://www.surveymonkey.com) in May-June 2015 to the 201 applicants who interviewed at CHofSA in its inaugural recruitment season, and in April-May 2016 to the 148 applicants who interviewed during the second recruitment season.

### Post-interview survey

The survey requested basic demographic information ( Table 1), including sex, medical degree, location of medical school (in state = Texas, out of state, or international medical school), number of interviews the applicant attended, number of programs the applicant ranked, and how the applicant ranked our program.

The applicants were then asked to rate specific program characteristics on a 7-point Likert scale as to the effect they had on the applicant’s ranking decisions for our program ( Tables 2 and 3). Program characteristics included items common to all programs, such as curriculum, benefits, and patient volume, as well as items specific to a new program such as lack of upper level residents, newness of the program, the opportunity to work one-on-one with faculty, and the opportunity to help shape the program. The survey also assessed items related to recruitment practices ( Table 4), including the vividness and early readiness of a program website, availability of a pre-interview season open house, and opportunities to meet with faculty during the interview day.

Additionally, a free response section on the survey solicited respondents to express their top concerns and motivators while considering this new program and the recruitment practices that did help or which would have helped to alleviate any concerns.

### Statistical analysis

For each Likert scale response, a Somers’ *D* directional coefficient was calculated based on how respondents ranked the program to determine the correlation between each characteristic or practice and an applicant’s likelihood of ranking the program highly. Program characteristics and recruitment practices were assigned a value of 1 (large or moderate positive effect), 2 (slight positive, no effect, or slight negative effect), or 3 (moderate or large negative effect) on ranking the program. The respondents’ rankings of the program were given a value of 1 (first preference), 2 (second preference), 3 (ranked between 3 and 5), 4 (ranked below 5), and 5 (not ranked). SAS software v9.4 (Cary, NC) was used to generate the Somers’ *D* values, 95% confidence intervals, and *p*-value for each survey item. We accepted two-sided α ≤ 0.05 as indicative of statistical significance.

### Qualitative analysis

Free text responses on the survey were analyzed by an online word cloud generator ( https://www.wordclouds.com) with the frequency of words within responses graphically represented in larger text to illustrate popularity. Questions included in this analysis were: (1) “What were the greatest attractive features of joining a new program?” (2) “What were your biggest concerns about joining a new program?” and (3) “What aspects of your interview day were most helpful in your evaluation of Baylor San Antonio as a new program?”

## Results/Analysis

In 2015, 72 of 201 (35.8%) surveys were returned by the first class of interviewees. In 2016, 91 of 148 (61.5%) surveys were returned by the second class of interviewees. The overall response rate was 163/349 (46.7%).

Table 1 describes respondent demographics. The majority were female (74%), graduating from an allopathic medical school (76%), and from a medical school in Texas (56%). A large majority of respondents reported interviewing at 5-15 pediatric residency programs (74%), and ranking 5-15 programs (77%) for the residency match. Twenty-five percent of respondents reported ranking the BCM-CHofSA residency program first or second for the match (i.e., “high rankers”), 25% ranked BCM-CHofSA between third and fifth (i.e., “intermediate rankers”), and 50% ranked the program 6 ^th^ or below, or not at all (i.e., “low rankers”).

**Table 1.** Demographics of survey respondents

Survey Item	N (%) ^1^		
	2015	2016	Total
**Sex**			
Female	55 (76)	66 (73)	121 (74)
Male	17 (24)	25 (27)	42 (26)
**Degree**			
MD	54 (75)	70 (77)	124 (76)
DO	11 (15)	21 (23)	32 (20)
MBBS	7 (10)	0 (0)	7 (4)
**Location of Medical School**			
In state (Texas)	33 (46)	58 (64)	91 (56)
Out of state	26 (36)	33 (36)	59 (36)
International	13 (18)	0 (0)	13 (8)
**Where did you rank Baylor-CHofSA?**			
1 ^st^	13 (19)	9 (10)	22 (14)
2 ^nd^	8 (11)	10 (11)	18 (11)
3 ^rd^ - 5 ^th^	25 (35)	16 (18)	41 (25)
Below 5 ^th^	22 (31)	49 (54)	71 (44)
Did not rank	3 (4)	7 (7)	10 (6)
**How many total programs did you rank?**			
Less than 5	5 (7)	3 (3)	8 (5)
5-10	20 (28)	27 (31)	47 (29)
10-15 ^2^	35 (49)	41 (46)	76 (48)
More than 15	11 (16)	18 (20)	29 (18)
**With how many programs did you interview?**			
Less than 5	3 (4)	3 (3)	6 (4)
5-10	17 (24)	21 (23)	38 (23)
10-15 ^2^	38 (53)	45 (50)	83 (51)
More than 15	14 (19)	22 (24)	36 (22)

^1^ Not all items were answered by all respondents; percentages are based on total responses to the corresponding survey item.

^2^ The online version of the survey inadvertently labeled the “11-15” response as “10-15,” causing the 5-10 and 10-15 categories to overlap. An individual whose answer was “10” to these questions may have chosen either response. CHofSA = The Children’s Hospital of San Antonio; DO = Doctor of Osteopathic Medicine; MBBS = Bachelor of Medicine, Bachelor of Surgery; MD = Doctor of Medicine.

Each program characteristic ( Tables 2 and 3) and recruitment practice ( Table 4) response in the survey is accompanied by a Somers’ *D* coefficient, 95% confidence interval, and *p*-value. A coefficient closer to 1 indicates that the item was more likely to be ranked positively by high rankers and negatively by low rankers. A coefficient closer to 0 indicates that the item exhibited poor correlation between positive and negative responses among high and low rankers. Negative coefficients suggest negative responses among high rankers and positive responses among low rankers; no statistically significant negative coefficients were observed.

Table 2 lists the survey items that assess program characteristics intrinsic to our new training program. All of these items showed significant positive correlations, indicating that they were considered more appealing program features to high rankers and more concerning to low rankers. Table 3 lists the survey items that assess characteristics not distinctive of a new training program. Most of these characteristics also showed significant correlations, although several program features did not have a significant correlation with ranking decisions. These less-impactful characteristics included: recommendation by residents and/or students at the home institution, recommendation by faculty mentor, intern night shift system, and the department chair.

**Table 2.** Impact of characteristics of a new program on applicant rank decision

Program Characteristics	Somers’ *D* (95% CI)	*p*-value
Opportunity to help shape the program *	0.580 (0.433 - 0.728)	<0.01
Newness of program *	0.515 (0.400 - 0.631)	<0.01
Opportunity to work one-on-one with faculty *	0.492 (0.325 - 0.659)	<0.01
Opportunity to be one of the first graduates *	0.483 (0.356 - 0.610)	<0.01
Lack of fellows in program *	0.406 (0.243 - 0.569)	<0.01
Lack of upper level residents *	0.337 (0.172 - 0.502)	0.01

* *p* ≤ 0.05

**Table 3.** Impact of characteristics not distinctive of a new program on applicant rank decision

Program Characteristics	Somers’ *D* (95% CI)	*p*-value
Advocacy opportunities *	0.517 (0.357 - 0.676)	<0.01
Size of program (number of residents) *	0.509 (0.385 - 0.633)	<0.01
Physical facilities *	0.508 (0.361 - 0.656)	<0.01
Proximity to family/friends *	0.474 (0.344 - 0.604)	<0.01
Program’s affiliation with Baylor College of Medicine *	0.447 (0.245 - 0.648)	<0.01
Research opportunities *	0.424 (0.258 - 0.591)	<0.01
Benefits other than salary *	0.423 (0.229 - 0.618)	0.02
Patient problems mix *	0.420 (0.253 - 0.587)	<0.01
Program’s emphasis on education *	0.413 (0.240 - 0.585)	<0.01
Recommendation by others outside your institution *	0.409 (0.150 - 0.667)	0.01
Patient volume *	0.405 (0.215 - 0.596)	0.01
Community hospital setting *	0.382 (0.217 - 0.546)	<0.01
Significant other’s job *	0.378 (0.214 - 0.542)	<0.01
Adaptive/individualized learning *	0.372 (0.195 - 0.548)	<0.01
Supplemental learning opportunities *	0.362 (0.178 - 0.547)	0.02
Credentials/experience of faculty *	0.357 (0.179 - 0.535)	0.01
Primary care educational curriculum *	0.355 (0.182 - 0.528)	<0.01
Experience of support staff with teaching environment *	0.342 (0.158 - 0.525)	<0.01
Overall educational curriculum *	0.319 (0.140 - 0.499)	<0.01
Board review curriculum *	0.316 (0.132 - 0.499)	<0.01
Resident space *	0.315 (0.141 - 0.490)	<0.01
Affiliation with an academic institution *	0.310 (0.108 - 0.512)	<0.01
Presence of a fourth year chief *	0.292 (0.108 - 0.476)	<0.01
Presence of other learners *	0.276 (0.081 - 0.470)	<0.01
Experience of program leadership *	0.275 (0.094 - 0.456)	<0.01
Salary *	0.274 (0.047 - 0.501)	0.02
Program Director *	0.251 (0.033 - 0.469)	0.03
Mentoring program *	0.239 (0.052 - 0.426)	0.01
Upper level resident call schedule *	0.238 (0.030 - 0.446)	0.03
Recommendation by residents and/or students at your institution	0.221 (-0.056 - 0.498)	0.13
Recommendation by faculty mentor	0.217 (-0.002 - 0.436)	0.06
Intern night shift system	0.193 (-0.005 - 0.391)	0.06
The city of San Antonio *	0.158 (0.017 - 0.300)	0.03
Department Chair	-0.029 (-0.231 - 0.173)	0.78

* *p* ≤ 0.05

The recruitment practices assessed in our survey are shown in Table 4. Although none of these practices are limited to new residency programs, several practices describing written and in-person communications related to the interview had significant positive correlations. We note that the strength of correlation (i.e., magnitude of Somers’ *D* coefficient) is lower among the recruitment practices than was seen among the most impactful program characteristics in Tables 2 and 3.

**Table 4.** Impact of recruitment practices on applicant rank decision

Recruitment Practices	Somers’ *D* (95% CI)	*p*-value
Follow up communication from program after interview *	0.308 (0.119 - 0.496)	<0.01
Written information available about program prior to interview season *	0.294 (0.099 - 0.488)	<0.01
Website vividness *	0.254 (0.054 - 0.454)	0.01
Email communication with program prior to interview season *	0.248 (0.049 - 0.447)	0.02
Opportunity to interview with program director *	0.235 (0.006 - 0.464)	0.05
Opportunity to attend open house prior to interview	0.210 (-0.037 - 0.457)	0.10
Opportunities to interact with faculty: Pre-interview evening event *	0.209 (0.012 - 0.407)	0.04
Opportunities to interact with faculty: Tours *	0.208 (0.012 - 0.404)	0.04
Website content	0.192 (-0.002 - 0.385)	0.05
Written materials available about program	0.188 (-0.016 - 0.392)	0.07
Opportunities to interact with faculty: Meet & Greet Luncheon	0.182 (-0.022 - 0.387)	0.08
Opportunity to rotate in hospital during fourth year	0.177 (-0.032 - 0.386)	0.10
Opportunity to observe an educational experience	0.167 (-0.033 - 0.368)	0.11
Flexibility of interview scheduling	0.144 (-0.049 - 0.336)	0.14
Escort by program staff throughout the day	0.143 (-0.055 - 0.341)	0.16
Online information about program available prior to interview season	0.133 (-0.066 - 0.331)	0.19
Opportunity to meet with department chair	0.064 (-0.141 - 0.269)	0.54
Interview day organization	0.030 (-0.175 - 0.236)	0.77
Opportunity to hear from chief resident	0.022 (-0.180 - 0.223)	0.83
Hotel provided	-0.045 (-0.269 - 0.178)	0.69
Interviewers who knew my application	-0.129 (-0.370 - 0.112)	0.30

* *p* ≤ 0.05

**Figure 1.**
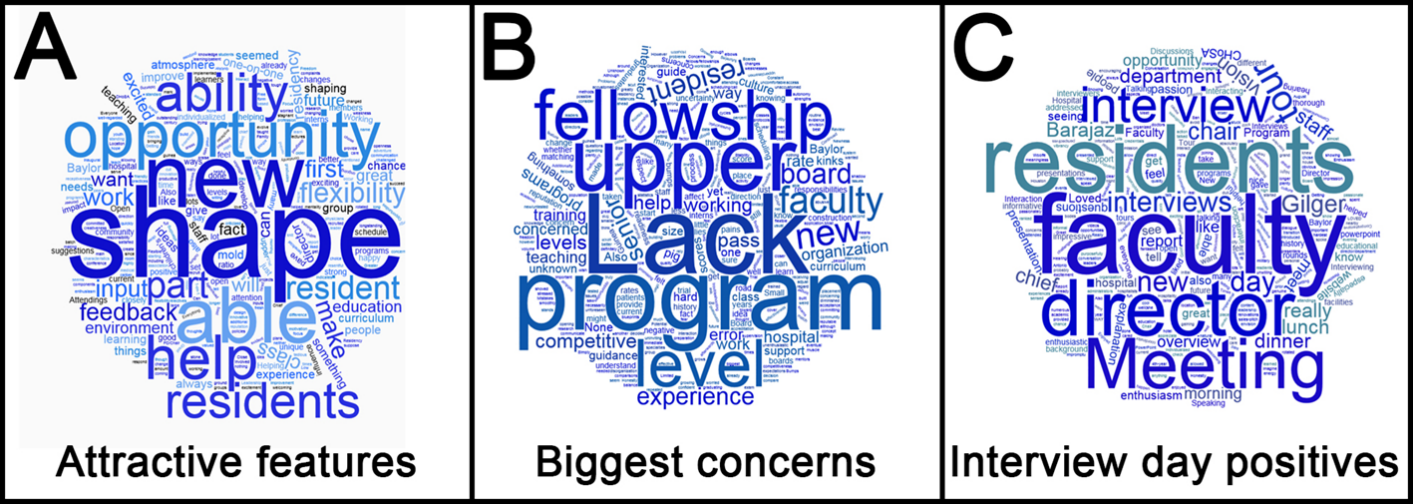
Word cloud analysis of free responses on survey. Larger text size correlates with frequency of the word’s use in the survey responses. A, responses to question, “What were the greatest attractive features of joining a new program?” based on 41 responses. B, responses to question, “What were your biggest concerns about joining a new program?” based on 43 responses. C, responses to question, “What aspects of your interview day were most helpful in your evaluation of Baylor San Antonio as a new program?” based on 38 responses.

The free responses in the survey were analyzed qualitatively by word cloud generation. The first question, “What were the greatest attractive features of joining a new program?” yielded 41 responses ( Figure 1A). Similar to the quantitative data ( Table 2), responses frequently included the desire to shape a new program and aid in its growth. By contrast, the second question, “What were your biggest concerns about joining a new program?” yielded 43 responses ( Figure 1B) and reflected an uncertainty about outcomes including fellowship match data, lack of upper-level residents, and experience of program faculty. To assess recruitment practices, the third question asked, “What aspects of your interview day were most helpful in your evaluation of Baylor San Antonio as a new program?” and yielded 38 responses ( Figure 1C). Reflecting some of the positive communication themes from the quantitative data ( Table 4), responses included the interviews and interactions with faculty and program leadership. Respondents to the 2016 (second year) survey also reported meeting with the first-year residents during the interview day as beneficial.

## Discussion

This study identified the characteristics of a new residency program and the recruitment practices that appear to have the greatest influence on applicant rank order. Although applicants have reported to NRMP the factors that are most important in determining their ranking decisions (NRMP, 2015), several of these factors cannot be measured in new programs. Conversely, certain characteristics unique to new programs are not included on the NRMP standard survey. Our survey results are the first to provide specific information on characteristics and recruitment practices that may make a new residency program attractive to applicants.

Several characteristics unique to a new program correlated highly with attracting certain applicants. They included helping to build a new program and working closely with faculty without the traditional learner hierarchy ( Table 2 and Figure 1A). Interestingly, the lack of senior residents was also listed as a chief concern by some respondents ( Figure 1B), illustrating one difference between applicants who might or might not be a good fit for a new program.

Our survey response rate (46.7%) from applicants to our program is comparable to the overall response rate of 47.5% reported nationally for the 2015 NRMP Applicant Survey (NRMP, 2015). Approximately 75% of our respondents interviewed at and ultimately ranked 5-15 programs ( Table 1), congruent with data of U.S. medical school seniors who matched to pediatrics in 2015 (median number of interviews/candidate = 12; median number of programs ranked/candidate = 11). We noted a higher response rate in the second recruiting year (62% in 2016 vs. 36% in 2015). We attribute this difference to sending the 2016 survey to interviewees four weeks earlier than we did in 2015, which may have influenced the likelihood of completing the survey.

Our study has several limitations. There may have been an association bias, such that respondents who were favorably disposed to our program may have answered all questions more favorably, and vice versa. It is difficult to assess this effect, although we note multiple non-significant results in Tables 3 and 4 that counter suspicion of this bias. We initially considered the possibility of response bias in our survey, such that applicants who ranked us higher would be more likely to respond. Instead, the observed result ( Table 1) was that 25% of the respondents ranked us first or second, whereas the remainder ranked us lower or not at all, indicating that our respondents were more broadly representative of the total pool of interviewees. Our findings represent a single new training program and may not directly generalize to all new programs. For example, one of the survey items specifically asked about our academic affiliation with BCM ( Table 3) and garnered a significant positive response. It is possible that applicants to our new program had different overall application patterns than those that applied only to established programs; our study was not designed to identify these potential differences. Since each established and new training program has inherent differences, and since nationally-available data does not adequately capture the qualities that could make new programs distinctively attractive to applicants, we share our findings with the intention of identifying themes that could be used to emphasize the value of new programs during recruitment.

## Conclusion

This study demonstrates that the unique qualities of a new program and communication-focused recruitment practices were attractive to a distinct subset of applicants to a new residency program. As the gap between applicants in the NRMP and available PGY-1 positions continues to widen, with 42,370 applicants for 27,860 positions in the 2016 match ( NRMP, 2016), the need for new residency training opportunities becomes more acute with each passing year. Our findings will help leaders of future new programs develop recruitment materials and interview practices to highlight the benefits of joining a new program, to address the concerns that applicants might have with the unproven nature of a new program, and to attract applicants who are the best fit for the opportunities and challenges of a new residency program. We look forward to more systematic evaluation of recruitment practices across new programs to help guide this important approach to solving the global healthcare workforce dilemma.

## Take Home Messages

• New residency programs are an essential tool to help meet projected physician workforce shortages, but face unique recruitment challenges.
• Certain factors related to a new program influence ranking decisions significantly.
• A subset of applicants may find attributes of a new program attractive.
• Communication-based recruitment practices can help identify and attract best-fit applicants to a new program.

## Notes On Contributors

Drs. Barajaz, Denniston, and Wolfe (ORCID 0000-0002-3113-2298) work in educational leadership for the Baylor Pediatric Residency Program at The Children’s Hospital of San Antonio. Ms. Shelley Kumar is a statistician and researcher for the Center for Research, Innovation, and Scholarship in Medical Education at Texas Children’s Hospital.
