# Supplementary material for: If You Build It, Will They Come? An Analysis of Candidate Attitudes Toward A New Residency Program
Source: MedEdPublish (2016). 2018 Nov 5;7:245. [Version 1] doi: 10.15694/mep.2018.0000245.1 (PMC10711978; doi:10.15694/mep.2018.0000245.1)
Supplement: Peer review history

# Reviewer report: Reviewer response for version 1

**DOI:** 10.21956/mep.19696.r29449

**Published:** 2018-11-22

**Authors:** David Bruce

**Affiliations:** 1 None

This review has been migrated. The reviewer awarded 4 stars out of 5 This is a well written paper which covers an increasingly important topic. I think it will have wide interest from all involved in recruiting to trading programs wither new or established. The study is well designed and presents its findings clearly. A wider review of studies looking at recruitment to medical programs in other healthcare systems would be helpful to readers. There are important messages for those setting up new programs about the importance of good communication and engagement with potential recruits, and making clear that they will have a role in shaping the program. The area that I felt was not covered in this paper was the importance of location. Previous studies have shown that location can can even trip choice of specialty when doctors are considering their careers. Perhaps this aspect could have been picked up in the discussion. This study has important findings and setting these in the wider literature would is paper

Reviewer Expertise:

NA

No decision status is available

# Reviewer report: Reviewer response for version 1

**DOI:** 10.21956/mep.19696.r29450

**Published:** 2018-11-13

**Authors:** Johnny Lyon Maris

**Affiliations:** 1 Southampton GP Education Unit

This review has been migrated. The reviewer awarded 4 stars out of 5 Thankyou for this submission I found it really interesting. New programmes attract different groups for differing reasons and this study helps us understand what those reasons may be. The text reads easily and although you bring in multiple tables you have made the article clear. The tables should make things clearer but table 1,3 and 4 are complex and not clear on quick view. I would suggest simplifying and grouping the areas so they have greater impact. Table 2 is simple. You do not need to asterisk and quote the p value, one or the other is fine. Figure 1 is very clear and has a good impact.Your discussion seems rushed and does not, to my mind, cover the key themes of the study.Overall this is an important piece of work for anyone implementing a new programme but could be re-written to simplify the results, expand the discussion and grouping of the themes.

Reviewer Expertise:

NA

No decision status is available

# Reviewer report: Reviewer response for version 1

**DOI:** 10.21956/mep.19696.r29447

**Published:** 2018-11-07

**Authors:** Permphan Dharmasaroja

**Affiliations:** 1 Faculty of Science, Mahidol University

This review has been migrated. The reviewer awarded 4 stars out of 5 The authors identified the characteristics of the new BCM pediatrics residency program and the recruitment practices that might influence the applicants’ ranking decision by using an online survey that involved items common to all programs and also those specific to the new program. Furthermore, an open-ended question section was additionally included in order to identify major concerns and motivators. The results provide useful information that could be applied to other residency training programs to recruit potential applicants. However, there are some points that the authors may be able to clarify prior to further implementation, as follows.1. The specific items asked in the survey regarding the new program should be clearly described. All programs that are claimed to be new usually have some new aspects. What does the term “newness of the program” mean? An example of “the opportunity to help shape the program” should also be given. These details will make the questions asked in the survey more objective, and the applicants who are interviewed will have a similar understanding.2. For the free responses in the survey, is it possible that the answers were influenced by the list of the characteristics of the new program provided in the section before, resulting in the responses being similar to the quantitative data?3. In the discussion section, in order to make the authors’ statement that the characteristics of the new program correlate highly with attracting a distinct subset of applicants more convincing, information about the applicant profiles should be provided and a correlation study should be conducted.

Reviewer Expertise:

NA

No decision status is available

# Reviewer report: Reviewer response for version 1

**DOI:** 10.21956/mep.19696.r29451

**Published:** 2018-11-05

**Authors:** Ariela Marshall

**Affiliations:** 1 Mayo Clinic

This review has been migrated. The reviewer awarded 4 stars out of 5 The authors have conducted an excellent survey-based study regarding applicant attitudes towards their new pediatrics residency program. They have identified several aspects that applicants reported had influenced their perception of the program and the way that they ranked the program, including positive aspects as well as potential negative aspects. The background section did a very nice job of discussing the reasons for creation of the program in an effort to expand the medical workforce.One concern is that – while I do not believe this is an official “match violation” per se – the ethics of asking exactly how applicants ranked a program are debatable. The survey was distributed after the match had already taken place so again does not technically constitute a match violation, but still raises some concern. One way to get around this may have been to separately survey applicants who did or not match to the program and see if there were differences in their responses without asking directly “how did you rank the program.”The discussion section is well done and the only point I would add is that the authors did not directly address the role of reputation of the program. Reputation was cited as a factor that has the potential to influence choice, which I certainly believe. However, the authors did not directly address the fact that the new residency program is at a medical center with an already stellar reputation (I was actually surprised to find that Baylor did not already have a pediatrics residency program) and this may have influenced the way interviewees perceived and ranked the program. Interviewing at a new program certainly brings the concerns cited by the authors/survey respondents, but I wonder if the reputation of the medical center itself may have mollified these concerns. It would be an interesting point for the authors to address.

Reviewer Expertise:

NA

No decision status is available

# Reviewer report: Reviewer response for version 1

**DOI:** 10.21956/mep.19696.r29448

**Published:** 2018-11-05

**Authors:** Mark Hanson

**Affiliations:** 1 Hospital for Sick Children

This review has been migrated. The reviewer awarded 4 stars out of 5 This paper speaks to a common and important matter for new residency programs (and new medical schools) and provides important insights for consideration when planning new recruitment and admissions processes. The authors’ use of work from Human Resources scholarship to inform their planning is innovative and something that should be utilized more broadly within our field. I believe an extension of their work that would be helpful to our readership is to explore the recruitment arc from application through to admissions interviews & offers and finally completion of the recruitment arc with enrollment data. As such I wonder if the authors could provide the full number of applicants and not only those who were asked to interviews and the program’s final enrollment yield (acceptances/admissions offers). Also, I think it would be interesting to know the proportion of Texas residents who accepted the program’s admissions offers as that is data that can inform a program’s recruitment strategy.

Reviewer Expertise:

NA

No decision status is available
